# Antidiabetic and Antioxidant Properties of Alkaloids from *Catharanthus roseus* (L.) G. Don

**DOI:** 10.3390/molecules18089770

**Published:** 2013-08-15

**Authors:** Soon Huat Tiong, Chung Yeng Looi, Hazrina Hazni, Aditya Arya, Mohammadjavad Paydar, Won Fen Wong, Shiau-Chuen Cheah, Mohd Rais Mustafa, Khalijah Awang

**Affiliations:** 1 Department of Chemistry, Faculty of Science, University of Malaya, Kuala Lumpur 50603, Malaysia; E-Mails: kingdom86_@hotmail.com (S.H.T.); hazrinahazni@um.edu.my (H.H.); 2 Department of Pharmacology, Faculty of Medicine, University of Malaya, Kuala Lumpur 50603, Malaysia; E-Mails: looicy@um.edu.my (C.Y.L.); aditya@um.edu.my (A.A.); paybarmj@gmail.com (M.P.); rais@um.edu.my (M.R.M.); 3 Department of Pharmacy, Faculty of Medicine, University of Malaya, Kuala Lumpur 50603, Malaysia; 4 Department of Medical Microbiology, Faculty of Medicine, University of Malaya, Kuala Lumpur 50603, Malaysia; E-Mail: wonfen@um.edu.my; 5 Faculty of Medicine and Health Sciences, UCSI University, No. 1, Jalan Menara Gading, UCSI Heights, Cheras 56000 Kuala Lumpur, Malaysia; E-Mail: cheahsc@ucsi.university.edu.my

**Keywords:** *Catharanthus roseus*, alkaloids, β-TC6, C2C12, cytotoxicity, antioxidant, glucose uptake, PTP-1B

## Abstract

*Catharanthus roseus* (L.) G. Don is a herbal plant traditionally used by local populations in India, South Africa, China and Malaysia to treat diabetes. The present study reports the *in vitro* antioxidant and antidiabetic activities of the major alkaloids isolated from *Catharanthus roseus* (L.) G. Don leaves extract. Four alkaloids—vindoline **I**, vindolidine **II**, vindolicine **III** and vindolinine **IV**—were isolated and identified from the dichloromethane extract (DE) of this plant’s leaves. DE and compounds **I**–**III** were not cytotoxic towards pancreatic β-TC6 cells at the highest dosage tested (25.0 µg/mL). All four alkaloids induced relatively high glucose uptake in pancreatic β-TC6 or myoblast C2C12 cells, with **III** showing the highest activity. In addition, compounds **II**–**IV** demonstrated good protein tyrosine phosphatase-1B (PTP-1B) inhibition activity, implying their therapeutic potential against type 2 diabetes. **III** showed the highest antioxidant potential in ORAC and DPPH assays and it also alleviated H_2_O_2_-induced oxidative damage in β-TC6 cells at 12.5 µg/mL and 25.0 µg/mL.

## 1. Introduction

*Catharanthus roseus* (L.) G. Don (Apocynaceae) is an ornamental shrub that grows up to 30–100 cm in height. It was previously known as *Vinca rosea* (L.) and commonly known as Madagascar periwinkle. Although this plant originated from Madagascar, it is widely distributed around the World due to its high survivability in a variety of habitats and use as an ornamental plant [1]. This plant has a long history as a folk medicine in many countries [2] such as South Africa, China, India, Mexico [3] and Malaysia [4], where it is utilized as a remedy to alleviate diabetes complications [5]. 

In Malaysia, the prevalence of known and newly diagnosed diabetes among adults of ≥30 years old showed an 80% rise from 8.3% in 1996 to 14.9% in 2006. In fact, Malaysia has already reached the projected prevalence for the year 2025. A survey by the Ministry of Health, Malaysia showed that majority of the diabetes patients were on oral medication (77%) and only a small percentage on insulin alone or in combination (7.2%). Apart from the standard treatment, some patients combined prescribed medications with alternative treatment, such as the use of local herbs, Chinese or Ayurvedic medicine. For example, some diabetes patients in Malaysia were reported to consume decoctions of this plant with improved health outcomes [6,7]. 

Most reports on antidiabetic activity of this plant have been conducted using crude extracts [8,9,10,11] rather than the pure bioactive compounds. Chattopadhyay and Singh *et al.* showed that the extract of this plant exhibited hypoglycemic activity in a streptozotocin induced diabetic rat model [12,13]. Ferreres *et al.* reported the scavenging ability for the aqueous leaves extract of this plant against DPPH (IC_50_ at 447 μg/mL), superoxide and nitric oxide radicals. Zheng *et al.* have showed the extract of this plant has the highest ORAC antioxidant capacity out of several medicinal herbs screened, with ORAC values of 22.30 μmol Trolox equivalent (TE)/g of fresh weight. The antioxidant activity of this plant was usually accessed along with their phenolic content as it exhibited high linear correlation with activity [14,15]. The antioxidant potential of alkaloids isolated from this plant has never been reported.

Our preliminary study has found that the dichloromethane leaves extract (DE) of *Catharanthus roseus* (L.) G. Don exhibited antidiabetic activity, with enhanced glucose uptake in pancreatic (β-TC6) and myoblast (C2C12) cells. Thus, the aim of this study is to provide scientific evidence by examining the hypoglycemic and antioxidant activity of alkaloids isolated from the active extract of this plant using biochemical and *in vitro* assays. Firstly, we evaluated the antioxidant potential of the isolated alkaloids using oxygen radical absorbance capacity (ORAC) and 1,1-diphenyl-2-picrylhydrazyl (DPPH) radical scavenging assays. Secondly, we determined the cell viability, glucose uptake and H_2_O_2_-induced intracellular reactive oxygen species (ROS) generation, followed by the evaluation of the PTP-1B inhibitory effects of the isolated alkaloids.

## 2. Results and Discussion

### 2.1. Results

#### 2.1.1. Identification of the Alkaloid Constituents

**Figure 1:**
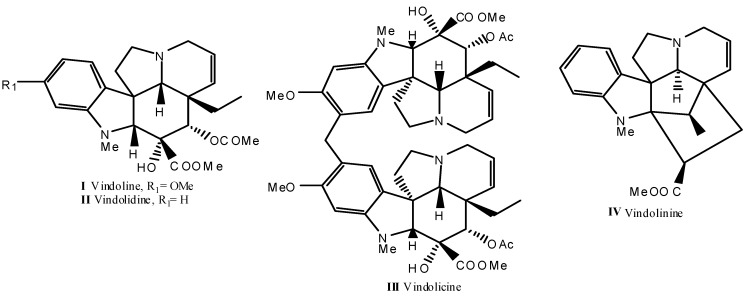
Chemical structures alkaloids **I**–**IV** isolated from leaves of *Catharanthus roseus*.

The study on the alkaloids of *Catharanthus roseus* (L.) G. Don through DE yielded four known indole alkaloids, namely vindoline **I**, vindolidine **II**, vindolicine **III**, and vindolinine **IV** (Figure 1). The structures of these known alkaloids were established by comparing their ^1^H, ^13^C-NMR, and mass spectral data with literature values [16,17,18,19]. The structures were further confirmed through evaluation of the 2D-NMR spectra; HSQC, HMBC and COSY. 

#### 2.1.2. Effect of Alkaloids on β-TC6 Cell Viability

The alkaloids were evaluated for cytotoxic activity on β-TC6 cells (Table 1). The cells were treated for 24 h with various concentrations of DE or alkaloids **I**–**IV**. Cell viability was determined by MTT assays. IC_50_ of DE and compounds **I**–**III** exceeded 50 µg/mL, while, compound **IV** showed the most potent inhibition, with an IC_50_ of 20.5 ± 3.6 µg/mL (Table 1). On the other hand, doxorubicin, the standard anticancer drug exhibited IC_50_ at 3.8 ± 1.7 µg/mL. The present study demonstrated for the first time that DE and compounds **I**–**III** are safe at high dosage, while **IV** showed a moderate cytotoxic effect. However, it must be noted that all alkaloids **I**–**IV** were at least five fold less toxic towards β-TC6 cells compared to doxorubicin.

**Table 1:** β-TC6 cell viability under treatment of DE and alkaloids **I**–**IV**.

Cytotoxicity (IC_50_)	DE	Vindoline	Vindolidine	Vindolicine	Vindolinine
µg/mL	78.4 ± 12.2	82.1 ± 9.8	76.7 ± 8.1	68.0 ± 10.4	20.5 ± 3.6
µM	-	180.1 ± 21.5	180.1 ± 19.0	73.5 ± 11.3	57.6 ± 10.7

#### 2.1.3. ORAC Evaluation

**Figure 2:**
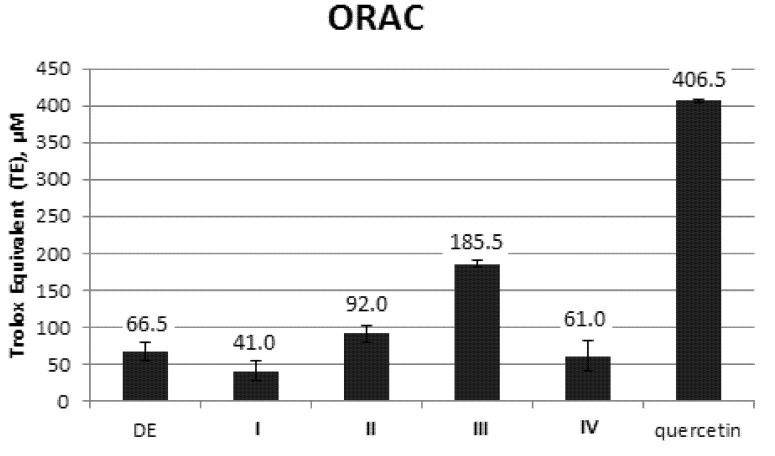
ORAC activity of DE and alkaloids **I**–**IV** isolated from the leaves of *Catharanthus roseus*. Quercetin is included as positive control.

The ORAC results were expressed as Trolox equivalent (TE). Net areas under the fluorescein decay curve (AUC) with increasing dosage of Trolox demonstrated a linearity correlation with R_2_ value 0.9668. Quercetin, the positive control demonstrated highest TE value at 406.5 ± 2.1 µM. Of all the isolated pure alkaloids, **III** (185.5 ± 4.9 µM) demonstrated the highest antioxidant property followed by **II** (92.0 ± 11.3 µM), **IV** (61.0 ± 19.8 µM) and **I** (41.0 ± 12.7 µM). DE also demonstrated relatively satisfactory activity at 66.5 ± 12.0 µM (Figure 2).

#### 2.1.4. DPPH Assay

**Figure 3:**
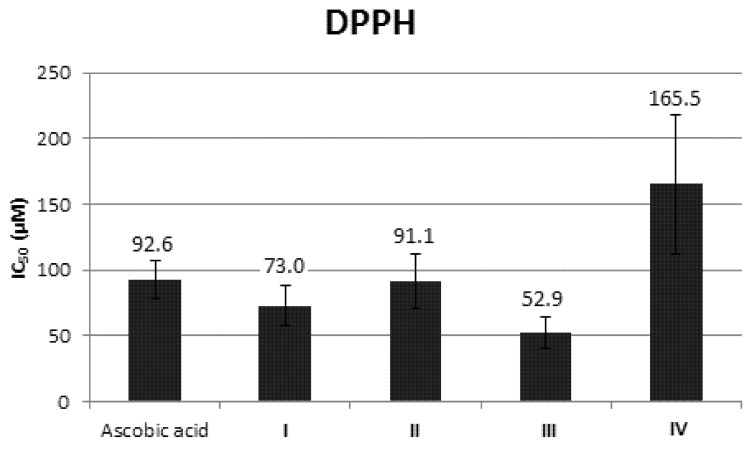
DPPH inhibition activity of alkaloids **I**–**IV**. Ascorbic acid is included as positive control.

DPPH inhibitory activity of the four alkaloids were evaluated and compared to ascorbic acid, as the positive control (Figure 3). Compound **III** exhibited the strongest DPPH inhibition activity (52.9 ± 12.0 μM), followed by **I** (73.0 ± 14.7 μM), **II** (91.1 ± 20.9 μM), and **IV** (165.5 ± 53.0 μM). Compounds **I**–**III** showed better DPPH radical scavenging ability than ascorbic acid. 

#### 2.1.5. Effect of Alkaloids on H_2_O_2_-induced ROS Production in β-TC6 Cells

**Figure 4:**
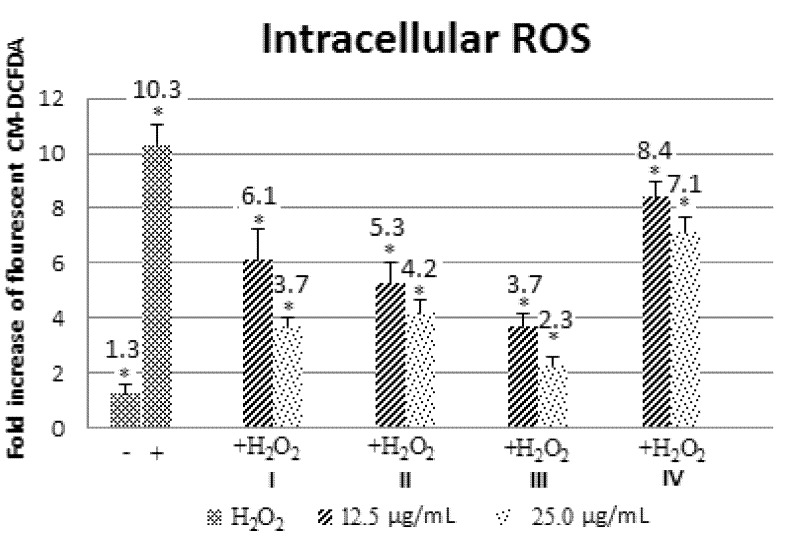
Intracellular H_2_O_2_-induced ROS production in β-TC6 cells pre-treated with alkaloids **I**–**IV** (* *p <* 0.05).

High ROS can cause oxidative stress in cells and antioxidants are important in controlling damage caused by ROS production. Next, we evaluate the efficacy of these alkaloids in reducing H_2_O_2_-induced oxidative stress by monitoring 5-(and-6)-chloromethyl-2',7'-dichlorodihydrofluorescein diacetate (CM-DCFDA) fluorescence. H_2_O_2_ treatment alone resulted in higher signal compared to control (Figure 4). Pre-treatment with all four alkaloids significantly decreased the oxidant burden in a dose-dependent manner (* *p* < 0.05; Figure 4). Among the four alkaloids, **III** showed the highest antioxidant protective effect, followed by **II**, **I** and **IV** in that order (Figure 4).

#### 2.1.6. Effect of Alkaloid on Glucose Uptake in β-TC6 and C2C12 Cells

**Figure 5:**
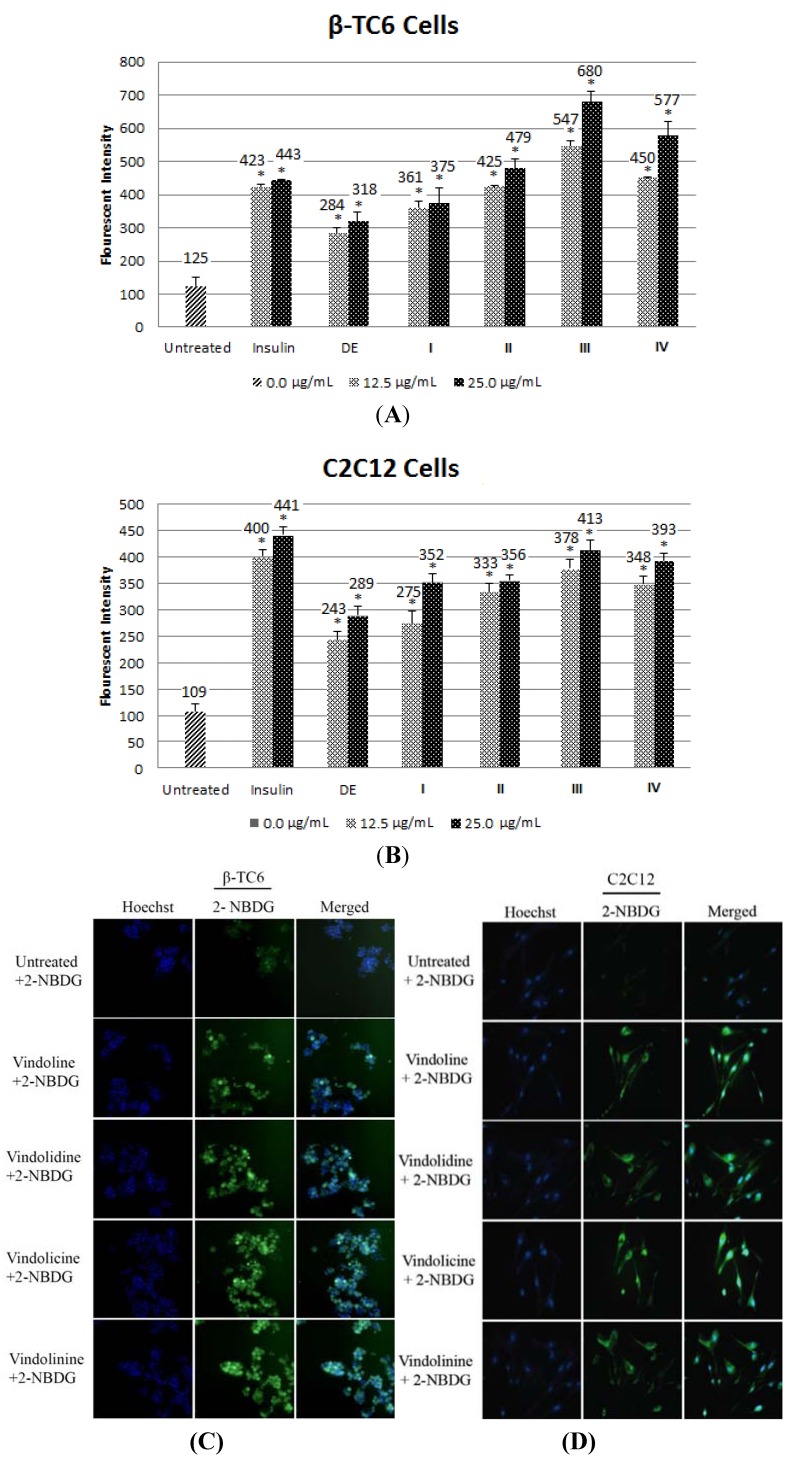
(**A**, **B**) Bar chart showing fluorescent intensity of 2-NBDG taken up by β-TC6 and C2C12 myoblast cells. Insulin was included as positive controls. (* *p <* 0.05) (**C**, **D**) Representative photos showing enhanced glucose uptake by β-TC6 and C2C12 after treated with 25 µg/mL of alkaloids **I**–**IV**. Blue:Hoechst, Green: 2-NBDG.

Improving glucose uptake in pancreatic or muscle cells could improve hyperglycemia condition of type 2 diabetes. DE dose-dependently increased glucose uptake, albeit the activity was relatively lower compare to the positive control, insulin (Figure 5A,B). Among the 4 pure alkaloids (**I**–**IV**) isolated, **III** stimulated the highest glucose uptake in β-TC6 and C2C12 cells with more than 3 fold enhancement in glucose uptake compared to untreated control (Figure 5A,B). Glucose clearance rates in **I**, **II**, and **IV** treated β-TC6 and C2C12 cells were significantly higher after treatment (Figure 5A,B). The fluorescent glucose 2-[N-(7-nitrobenz-2-oxa-1,3-diaxol-4-yl)amino]-2-deoxyglucose (2-NBDG) could be visualized around cytosol in β-TC6 and C2C12 cells treated with DE and the alkaloid **I**–**IV** (Figure 5C,D).

#### 2.1.7. Effect of Alkaloid on PTP-1B Inhibition

**Figure 6:**
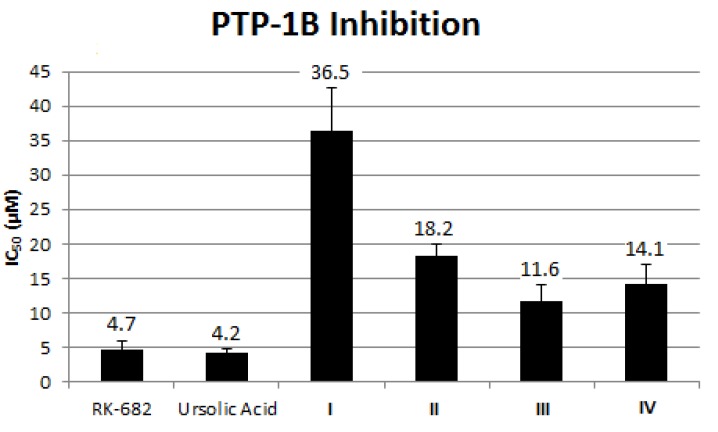
PTP-1B inhibition of isolated alkaloids **I**–**IV** compare against the positive control drugs RK-682 and ursolic acid.

The development of novel pharmaceutical agents that help ameliorate insulin resistance will be potentially important for the prevention and treatment of diabetes. PTP-1B is an enzyme that belongs to the protein tyrosine phosphatase family and a negative regulator of the insulin signaling pathway. To further evaluate antidiabetic potential of these compounds, we performed *in vitro* PTP-1B inhibition assays to determine whether DE or the isolated alkaloids **I**–**IV** were active inhibitors of PTP-1B. Compound **III** showed the highest inhibition activity, followed by **IV**, **II** and **I**. However, the activity of **III** was relatively weaker as compared to the positive controls (*R*)-3-hexadecanoyl-5-hydroxymethyltetronic acid (RK-682) and ursolic acid (Figure 6).

### 2.2. Discussion

In our study, the DCM leaves extract (DE) of this plant exhibited antioxidant and hypoglycemic activity in β-TC6 mouse pancreatic cells. Moreover, DE treatment resulted in good glucose uptake in C2C12 myoblast. The chemical investigation of DE yielded four alkaloids: vindoline **I** (1.0%), vindolidine **II** (0.14%), vindolicine **III** (0.07%) and vindolinine **IV** (0.02%). Compound **III** indicated the highest antioxidant property, followed by **II**, in our ORAC and DPPH assays. All four alkaloids exhibited moderate free radical scavenging activities. However, the presence of secondary reactions including those associated with repair mechanism [20] or antioxidant-metal reactions in ORAC assay may result in the reduction of active concentration which leads to under-estimation of ORAC value [21]. DPPH˙ is a relatively more stable radical which could provide a proper evaluation of a compound’s antioxidant activity [22]. In this study, we found that DPPH inhibition activity of **I**–**III** were better than the standard, ascorbic acid, except for **IV**. Overall, all four alkaloids exhibited relatively good antioxidant potential, with **III** being the best, although there are some slight discrepancies in the activity trends based on ORAC and DPPH assays.

This is the first report on hypoglycemic activity of **II** and **III** as Svoboda had previously reported slight and moderate *in vivo* hypoglycemic activity of **I** and **IV**, respectively [23]. All four alkaloids **I**–**IV** exhibited enhanced glucose uptake with dose dependent activity in β-TC6 and C2C12 cells. The lower dosage could provide antioxidant effect to β-TC6 cells, which promoted cellular activity, and led to an increased level of glucose uptake. Among the four alkaloids, **III** showed the greatest enhancement of glucose uptake in β-TC6 and C2C12 cells. At the lowest dosage (12.5 µg/mL), the degree of glucose uptake increased in the order of **I**, **II**, **IV** and **III**, correlating with its PTP-1B inhibition activity. PTP-1B is a negative regulator of the insulin signaling pathway in human and is considered a promising potential therapeutic target for treatment of type 2 diabetes. For example, PTP-1B knock-out mice showed hyper obesity and prone to the development of type-2 diabetes. Thus, in light of these evidences, we speculate that PTP-1B signaling could play a role in controlling cellular glucose uptake activity in both β-TC6 and C2C12 cells.

Type I diabetes develops through autoimmune destruction of islet β-cells, while dysregulation of insulin secretion from β-cells due to oxidative damage could lead to type 2 diabetes [24]. Islet β-cells are vulnerable to oxidative stress and have a poor DNA repair capacity against oxidative damage. Recent study showed that type 2 diabetic individuals exhibited imbalance of ROS generation and neutralization [25]. Oxidative stress by ROS plays a central role in the development of diabetic complication, insulin resistance and β-cells dysfunction [26]. In the present study, H_2_O_2_ was applied to induce OH˙ free radicals, one of the most reactive and destructive oxidants. Compound **III** showed the highest reduction of H_2_O_2_-induced ROS production in β-TC6 cells, followed by **II**, **I** and **IV**, indicating the potential of **III** in alleviating oxidative stress in pancreatic cells.

The present findings indicated that compound **III** possessed the best antioxidant and hypoglycemic activity through evaluation of ORAC, DPPH, ROS production, glucose uptake and PTP-1B inhibition assay compared to the other three alkaloids. Thus, we speculate that **III** is more beneficial and useful, in comparison with **I** or **II** or **IV**, for amelioration of type 2 diabetes due to its high antioxidant and PTP-1B inhibition activities. This strongly gives evidence to the use of this plant as an effective treatment of diabetes in several parts of the world and **III** can be viewed as a lead compound for the development of antidiabetic therapeutics.

## 3. Experimental

### 3.1. Plant Material

*Catharanthus roseus* (L.) G. Don. was cultivated at Jeli, Kelantan, Malaysia from November 2008 under natural condition. The leaves were collected around May 2009 and dried at 40 °C. The specimen was authenticated by Mr. Teo Leong Eng, a botanist in the Faculty of Science, University of Malaya. A voucher specimen with Herbarium No. of KL 5763 was deposited at the Herbarium in Department of Chemistry, University of Malaya, Kuala Lumpur, Malaysia. 

### 3.2. Extraction and Fractionation

Dried grounded leaves of this plant (1 kg) was macerated with *n*-hexane (hex, 10.0 L) for 3 days at room temperature and this process was repeated twice. After removal of solvent, the plant residue was first wetted with 25% ammonia for an hour, followed by soaking with dichloromethane (DCM). The DCM extract (DE) was obtained after filtered and dried under reduced pressure. DE (42.6 g) was subjected for acid-base extraction using 5% hydrochloric acid (HCl) and 25% ammonia solution to obtain 4.2 g of alkaloid crude (DA).

DA was subjected to column chromatography (CC) with silica gel 60 (Merck, Darmstadt, Germany) using gradient elution from DCM (100%) and DCM-methanol (95:5, 90:10, 85:15, 80:20, 75:25, 70:30, 65:35, 60:40, 55:45, 50:50, v/v). The column was later flushed using DCM-methanol (40:60, 20:80, v/v) and methanol (100%). 1000 mL of each different solvent system were used for elution. The eluent was collected in fractions of 100 mL. The fractions with the same thin layered chromatography (TLC) profile were then combined. A total of 15 fractions were obtained (Fractions 1–15).

### 3.3. Isolation and Purification

Fraction 3 was subjected to preparative TLC (PTLC) separation using hex-ethyl acetate-acetone (20:78:2, v/v/v) under ammonia vapour on TLC silica gel 60 F_204_ (Merck) to yield **I** (428.1 mg) and **II** (57.9 mg). Fraction 4 was applied to PTLC with hex-ethyl acetate-acetone (20:79:1, v/v/v) under ammonia vapour to yield **III** (31.0 mg). PTLC of fraction 6 using the same solvent system as applied on fraction 4 yielded **IV** (8.4 mg). 

### 3.4. Identification and Characterization of Alkaloids

One-dimensional and two-dimensional of ^1^H and ^13^C-NMR experiments were carried out on JEOL ECA 400 FT-NMR. CDCl_3_ was used as the solvent. The chemical shifts were recorded with reference to CDCl_3_ (7.24 ppm). LC-MS identification was carried out on Shimadzu Liquid Chromatography Mass Spectrometry-Ion Trap-Time Of Flight system equipped with pump (LC-20AD), autosampler (SIL-20AC), column oven (CTO-20AC), PDA detector (SPD-M20A) and coupled to an ESI interface (Shimadzu, Kyoto, Japan). 5 µL of isolated compounds were injected and chromatographed on a Waters XBridge C18 2.5 mm (2.1 × 50 mm) column with the mobile phase of A (0.1% formic acid in water) and B (0.1% formic acid in methanol). The mobile phase was performed in a step gradient as follows: isocratic 10% B for two min, 10% B to 100% B over the next 25 min and isocratic at 100% B for 5 min. The system was re-equilibrated for 5 min before the next run. Eluent were monitored using diode array detector at 220 nm, 254 nm, 350 nm and 450 nm. ESI-MS was performed in the range 100–2,000 in both the positive and negative mode. The temperature of the heat block and curved desolvation line (the inlet for the high vacuum region) were set to 200 °C and 250 °C, respectively. Nitrogen gas was used as nebulizer with the flow rate set at 1.5 L/min. The ESI source voltage was set at 4.5 kV for positive mode and −3.5 kV for the negative mode whereas the detector was maintained at 1.7 kV. Shimadzu’s LCMS solution software was used for data analysis.

### 3.5. Cell Culture

Mouse β-TC6 pancreatic cell line and mouse myoblast (skeletal muscle) C2C12 cell line were purchased from American Type Culture Collection (ATCC, Manassas, VA, USA). β-TC6 was cultured in 15% fetal bovine serum (FBS) in Dulbecco’s Modified Eagle Medium (DMEM) [27]. C2C12 cells were maintained in DMEM supplemented with 10% FBS. The cultures were kept in a humidified incubator at 37 °C in 5% CO_2_ and the growth medium was changed every 3 days.

### 3.6. Cellular Viability

The cytotoxic effects of DE and alkaloids on β-TC6 cell growth was determined by an MTT assay [28,29]. Briefly, 1.5 × 10^4^ cells were seeded into a 96-well plate and incubated at 37 °C in 5% CO_2_ for 24 h. The second day, the seeded cells were treated with alkaloids and DE. After another 24 h of incubation, MTT solution was added at 2 mg/mL and the plates were kept in incubator at 37 °C in 5% CO_2_ for 1 h. Absorbance at 570 nm was measured by a Plate Chameleon V microplate reader (Hidex, Turku, Finland). The obtained values are expressed as a percentage of cell viability after exposure to DE and alkaloids for 24 h. Cell viability was defined as the ratio (percentage) of the absorbance of treated cells to that of untreated ones.

### 3.7. ORAC Assay

The ORAC assay was carried out based on the previously described procedure with slight modifications [30,31]. In brief, the sample/blank (175 μL) was dissolved in phosphate buffer saline (PBS) at the concentration of 160 μg/mL at pH 7.4. The Trolox standard was prepared in serial dilutions starting from 75 mM. Standard 96-well black microplates was used for the assay, and 25 μL each of the samples (DE and alkaloids), standard (Trolox), blank (solvent/PBS), or positive control (quercetin) were added to the wells. Fluorescent sodium salt solution was added at 150 μL per well, followed by incubation at 37 °C for 45 min. The total volume of each well was made up to 200 μL by adding 2,20-azobis-(2-amidinopropane) dihydrochloride (AAPH) solution. Fluorescence value was recorded at 37 °C until it became 0 (excitation at 485 nm, emission at 535 nm) using a fluorescence spectrophotometer (Perkin-Elmer LS 55) equipped with an automatic thermostatic autocell-holder. Data were collected every 2 min for 2 h and the data analysis was subsequently done by calculating the differences of AUC between the blank and the sample. The resulted values are expressed as Trolox equivalents.

### 3.8. DPPH Assay

The scavenging activity of all 4 alkaloids on DPPH (1,1-diphenyl-2-picrylhydrazyl) was determined based on the reduction of purple DPPH to yellow coloured diphenylpicrylhydrazine [32]. Different concentrations of the alkaloids in ethanol (ranging from 10–600 μg/mL) were tested. Sample solution in different concentrations was added at 2.5 mL in each well. Next, 0.3 mM DPPH ethanol solution (1 mL) was added to produce the test solutions, while ethanol (1 mL) was added to produce the blank solutions. Another 1 mL of DPPH solution was mixed with ethanol (2.5 mL) to prepare the negative control. The solutions were kept in the dark at room temperature for 30 min to let them react. The measurement of absorbance and colour changes was done at the wavelength of 518 nm. The following equation was applied to convert the absorbance values into percentage antioxidant activity:

% Inhibition = [(B − T)/B] × 100

where B: absorption of blank sample; T: absorption of tested samples. The half maximal inhibitory concentration (IC_50_) and the kinetics of DPPH scavenging activity were determined. Ascorbic acid was used as positive control in this assay.

### 3.9. Intracellular ROS Measurement

The fluorescent probe, CM-DCFDA (Invitrogen, Carlsbad, CA, USA), was used to monitor the intracellular generation of reactive oxygen species (ROS) by H_2_O_2_, as previously described [33]. In brief, β-TC6 cells were pretreated with compounds for 2 h. CM-DCFDA (20 μM) was added for additional 30 min. The medium was then replaced with fresh medium containing 250 μM H_2_O_2_ and the cells were incubated for another 20 min. The intracellular ROS were determined using a fluorescence spectrophotometer at Excitation/Emission = 485 nm/530 nm (Synergy Neo, Bio-Tek, Winooski, VT, USA). 

3.10. 2-NBDG Glucose Uptake

β-TC6 or C2C12 cells were seeded at a density of 1.5 × 10^4^ cells/mL in 96-well plate and allowed to attach, spread, and proliferate to near confluence at 37 °C in 5% CO_2_. After overnight incubation, the medium was discarded, washed with phosphate-buffered saline (PBS) twice and replenished with 2.5 mM glucose in basal medium comprising DMEM without glucose or pyruvate supplemented with l-glutamine and 15% (v/v) FBS (final serum glucose concentration of approximately 0.25 mM). The cells were incubated for 60 min at 37 °C in 5% CO_2_. The conditioning medium was then replaced with 10 mM 2-NBDG (Invitrogen) in basal medium in the presence or absence of DE and alkaloids. The cells were kept at 37 °C in 5% CO_2_ for 30 min to permit endocytosis of the 2-NBDG. Then, the medium was removed, cells were washed twice with PBS and stained with nucleic dye Hoechst 33342 for another 30 min. The cells were then observed for intra-cellular fluorescence at *Excitation*/*Emission* = 350 nm/461 nm and Excitation/Emission = 475 nm/550 nm for Hoechst 33342 and 2-NBDG, respectively. Plates were evaluated using the ArrayScan High Content Screening (HCS) system (Cellomics Inc., Pittsburgh, PA, USA) and analyzed with Target Activation BioApplication software (Cellomics Inc.) [34].

### 3.11. PTP-1B Inhibition

*para*-Nitrophenyl phosphate (pNPP) was used as substrate to assay the phosphatase activity [35]. The ionic strength of the assay buffer (pH 7.4), was adjusted to 0.15 M using NaCl. The buffer contains 50 mM 3,3-dimethylglutarate, 1 mM ethylenediaminetetraacetic acid (EDTA), 5 mM glutathione, and 0.5% FCS (not heat inactivated). Briefly, diluted (50 μM alkaloids) and undiluted inhibitors were added to the reaction mixture containing 0 or 2.5 mM pNPP to reach the total volume of 100 μL. The enzyme (recombinant PTP-1B) was added to initiate the reaction. The reaction was allowed to proceed for 5 min before adding the inhibitor. The incubation was continued at 37 °C for 5–60 min and the time was recorded. Then 0.5 M NaOH in 50% ethanol (20 μL) was added to stop the reaction. To determine the enzyme activity, the absorbance was measured at 405 nm using a Tecan ELISA reader with appropriate corrections for absorbance of substrate, alkaloids, and nonenzymatic hydrolysis of substrate.

### 3.12. Statistical Analysis

Data were presented as means ± SEM (standard error of mean) and analyzed with unpaired Student’s *t*-test. *p <* 0.05 is considered significant.

## 4. Conclusions

In conclusion, all four alkaloids induced relatively high glucose uptake in β-TC6 and C2C12 cells. At low dosages, these alkaloids showed good antioxidant potential by alleviating H_2_O_2_-induced oxidative damage in β-TC6 cells. All four alkaloids; **I**–**IV** were less cytotoxic than the standard anticancer drug doxorubicin towards β-TC6 cells. Among the alkaloids, **III** demonstrated the most potent activity in PTP-1B inhibition, which supports further development of **III** as a novel PTP-1B inhibitor that may serve as “insulin sensitizer” in the management of type 2 diabetes.
